# Clinicopathological Associations of Plasma Cell‐Rich Rejection in Pediatric Liver Transplant Recipients

**DOI:** 10.1111/petr.70403

**Published:** 2026-07-14

**Authors:** R. Gemell, C. Hajinicolaou, M. Hale, P. Walabh

**Affiliations:** ^1^ Department of Paediatrics and Child Health University of Witwatersrand, Faculty of Health Sciences Johannesburg South Africa; ^2^ Division of Paediatric Gastroenterology, Department of Paediatrics and Child Health, Hepatology and Nutrition Chris Hani Baragwanath Academic Hospital Johannesburg South Africa; ^3^ Department of Anatomical Pathology, School of Pathology, Faculty of Health Sciences University of the Witwatersrand Johannesburg South Africa; ^4^ Division of Paediatric Gastroenterology/Hepatology and Nutrition, Department of Paediatrics and Child Health Charlotte Maxeke Johannesburg Academic Hospital Johannesburg South Africa

**Keywords:** acute cellular rejection, antibody‐mediated rejection, chronic rejection, medication level variability index, pediatric liver transplantation, plasma cell‐rich rejection

## Abstract

**Introduction:**

Liver transplantation is the standard treatment for pediatric patients with cirrhosis, acute liver failure, metabolic liver disease, and malignancy. Plasma cell‐rich rejection is a poorly characterized form of late‐onset rejection. We aimed to evaluate the incidence and clinicopathological factors associated with plasma cell‐rich rejection in pediatric liver transplant recipients in Johannesburg, South Africa.

**Methods:**

We conducted a retrospective review of 91 pediatric liver transplant recipients (≤ 18 years) who underwent transplantation between January 1, 2015, and December 31, 2021. Demographics, clinical characteristics, episodes of acute cellular rejection, Banff Rejection Activity Index scores, histopathological findings, medication level variability index, and outcomes were analyzed. Survival outcomes were assessed using the Kaplan–Meier methods and Cox proportional hazards models, with follow‐up censored as of 31 December 2023.

**Results:**

Of 91 recipients, 45 (49.5%) developed acute cellular rejection, with a median medication level variability index of 2.05 (1.1–3.3). Late‐onset acute cellular rejection occurred in 19 of these 45 patients (42.2%). Plasma cell‐rich rejection was identified in 14 patients (15.3%) and was associated with increased frequency and severity of acute cellular rejection episodes (*p* < 0.001), autoimmune seropositivity (*p* = 0.008), central perivenulitis (*p* < 0.001), and chronic rejection (*p* = 0.006). In multivariable logistic regression, recurrent rejection episodes (OR 22.04, 95% CI 5.08–95.52; *p* < 0.001) and severe rejection (OR 3.60, 95% CI 1.66–7.78; *p* = 0.001) were independently associated with PCRR. Plasma cell‐rich rejection was not independently associated with mortality in a Cox proportional hazards analysis (HR 0.51, 95% CI 0.15–1.73; *p* = 0.282). Mortality was independently associated with higher medication level variability index values (HR 1.06, 95% CI 1.03–1.09; *p* < 0.001), severe rejection Banff Rejection Activity Index score 7–9 (HR 1.42, 95% CI 1.00–1.99; *p* = 0.049), and cytomegalovirus disease (HR 3.71, 95% CI 1.67–8.25; *p* = 0.001).

**Conclusion:**

Plasma cell‐rich rejection is associated with frequent and severe rejection episodes, autoimmune markers, central perivenulitis, and chronic rejection. PCRR was not independently associated with mortality; mortality was associated with higher medication level variability index values, severe rejection, and CMV disease. Early recognition of these clinicopathological associations may support closer monitoring and inform future research into targeted management strategies.

## Graphical abstract

Plasma cell‐rich rejection (PCRR) occurred in 15.3% of pediatric liver transplant recipients. PCRR was associated with recurrent rejection, severe rejection, central perivenulitis, ANA positivity, and chronic rejection. PCRR was not independently associated with mortality.

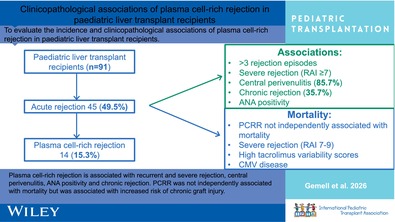

## 1 Introduction

Liver transplantation (LT) is the gold standard for pediatric patients with acute liver failure, chronic liver failure, metabolic liver disease, and liver tumors [1]. Despite improvements in immunosuppressive regimens, surgical techniques, critical care, and patient selection, acute cellular rejection (ACR) remains a major complication leading to graft loss and patient death if not promptly identified and managed [2]. ACR post‐LT presents with non‐specific signs and symptoms such as liver dysfunction and jaundice, making a liver biopsy the most reliable and definitive diagnostic tool [2].

The liver is considered an immunologically privileged organ, as evidenced by lower ACR rates than other solid organs, owing to its ability to induce immune tolerance [3]. Historically, liver allograft rejection was thought to be predominantly T‐cell‐mediated, with occasional cases of antibody‐mediated rejection (AMR) reported [4]. Previous research has introduced the concept of AMR, characterized by donor‐specific antibodies (DSAs) and complement activation (C4d), highlighting the need for new diagnostic criteria. In recent years, it has become evident that both T‐cell and antibody‐mediated mechanisms can mediate liver allograft rejection [5].

Formerly known as *de novo* autoimmune or plasma cell hepatitis, plasma cell‐rich rejection (PCRR) is a poorly described, infrequent form of late‐onset rejection (> 6 months post‐LT), with a reported incidence of approximately 3%–5% in liver transplant recipients [6]. The etiology of PCRR remains unclear, with ongoing debate regarding whether PCRR represents a subtype of AMR or a distinct pathological entity [5]. PCRR likely involves both T‐ and B‐cell‐mediated mechanisms, and has been associated with chronic rejection (CR) and graft failure [7].

Reported risk factors for PCRR include viral infections, poor adherence to immunosuppressive therapy, and recurrent ACR episodes [8, 9]. Poor adherence, in particular, has been linked to both early and late‐onset acute rejection episodes, with the medication level variability index (MLVI) serving as a surrogate marker for assessing adherence to immunosuppressive therapy [10]. There is currently no consensus on the optimal management of PCRR, with treatment protocols varying across transplant centers [8]. Strategies range from induction therapy with short‐course intravenous corticosteroids followed by a transition to oral steroids to the use of biologic agents such as anti‐thymocyte globulin (ATG) in steroid‐resistant cases [11]. Maintenance immunosuppression typically includes calcineurin inhibitors (CNI) such as tacrolimus, antiproliferative agents, and mammalian target of rapamycin (mTOR) inhibitors. In select cases, monoclonal antibodies such as rituximab have also been employed [9, 12, 13].

While PCRR has been documented in adult liver transplant recipients, it is poorly described in pediatric populations [7]. To our knowledge, limited data are available on PCRR in pediatric liver transplant (PLT) patients in low‐ and middle‐income countries. We aimed to analyze the incidence and clinicopathological associations of PCRR in a cohort of PLT recipients.

## 2 Methods

### 2.1 Study Design and Population

**FIGURE 1:**
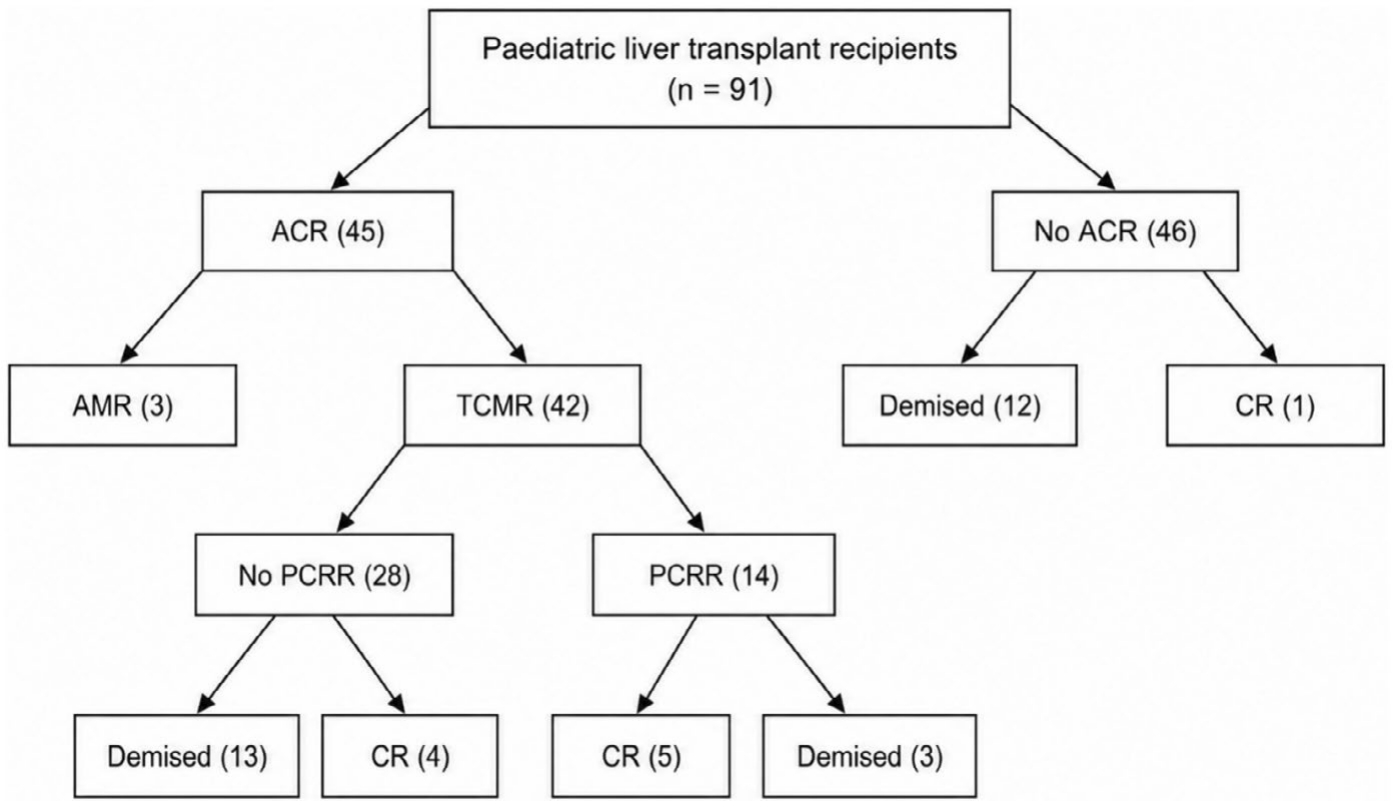
Flow diagram showing progression and outcomes of rejection types in pediatric liver transplant recipients. ACR; Acute cellular rejection, AMR; Antibody‐mediated rejection, CR; Chronic rejection, PCRR; Plasma cell‐rich rejection, TCMR; T‐cell mediated rejection.

We conducted a retrospective cohort study reviewing the medical records of 91 public‐sector patients under 18 years old who received liver transplants at the Wits Donald Gordon Medical Centre (WDGMC) between 1 January 2015 and 31 December 2021 and were followed up at the Charlotte Maxeke Johannesburg Academic Hospital (Figure 1). Inclusion criteria included all pediatric patients who received liver transplants at the WDGMC during the study period. Patients were excluded if they had incomplete medical records, a prior diagnosis of autoimmune hepatitis (AIH), or a history of re‐transplantation. Ethical approval for the study was obtained from the CMJAH Chief Executive Officer and the University of the Witwatersrand Human Research Ethics Committee (Ethics Clearance number: M2211108).

### 2.2 Study Procedures

Post‐transplant immunosuppression followed a standardized protocol. Induction therapy with methylprednisolone at a dose of 10 mg/kg was administered at the time of graft reperfusion. This was followed by a tapering regimen of intravenous corticosteroids: 1 mg/kg daily during the first week, 0.5 mg/kg daily in the second week, and 0.25 mg/kg daily for the subsequent three months. Once oral intake was tolerated, patients were transitioned to oral prednisone, which was discontinued after three months unless there were recent ACR episodes. Tacrolimus was initiated on day one post‐liver transplantation, with dosing adjusted to maintain target trough levels of 10–14 ng/mL during the first month, 8–10 ng/mL over the next two months, and 4–8 ng/mL thereafter.

### 2.3 Histopathology

Suspicion of ACR was based on biochemical and clinical indicators and confirmed by liver biopsy. All biopsies were reviewed at the National Health Laboratory Service (NHLS) and evaluated using the Banff Rejection Activity Index (RAI). A RAI score of greater than 7–9 was considered indicative of severe rejection, necessitating escalation of immunosuppressive therapy.

### 2.4 Management of Acute Cellular Rejection

Management of moderate to severe ACR included high‐dose methylprednisolone (10 mg/kg) pulsing for 3 days followed by a tapering course of prednisone, along with adjustment of tacrolimus levels. ATG was reserved for steroid‐resistant cases. Further immunosuppressive modifications were made based on the recurrence of ACR, the presence of donor‐specific antibodies (DSAs), or histological features of plasma cell‐rich rejection (PCRR).

### 2.5 Definitions

Key definitions used in this study are provided in (Table S1), and a list of abbreviations is provided in Table S2.

### 2.6 Statistical Analysis

We performed all statistical analyses using Stata version 17. Categorical variables were described using frequencies and percentages, while continuous variables were summarized as means with standard deviations or medians with interquartile ranges, depending on their distribution. Comparisons of categorical variables were made using the chi‐squared test, and continuous variables were analyzed using the independent samples *t*‐test. A *p*‐value ≤ 0.05 was considered indicative of statistical significance. Chronic rejection and patient survival were assessed using the Kaplan–Meier method and Cox proportional hazards model. For survival analyses, follow‐up was censored on 31 December 2023. Multivariable Cox proportional hazards regression was used to identify independent predictors of mortality, and hazard ratios (HRs) with 95% confidence intervals were reported.

## 3 Results

### 3.1 Patient Demographics and Baseline Characteristics

Of the 91 PLT recipients, 39 (42.8%) were male. The median age at transplantation was 29.6 months (IQR 62.8). Biliary atresia (BA) was the most common underlying cause for transplantation (Table 1), affecting 46 patients (50.5%), followed by acute hepatitis A, which affected 12 patients (13.2%). The majority of recipients were Black, 72 (79.2%). More than half of the transplants (49; 53.8%) were from related living donors (RLD), with the remainder being deceased‐donor liver transplants (Table 1).

**TABLE 1:** Demographic and clinical characteristics of study patients.

Characteristics	*N* (%) or median IQR
Age at transplant (months), median (IQR)	29.6 (62.8)
Weight at transplant (kg), median (IQR)	13.2 (11.5)
**Sex *n* (%)** Male Female	39 (42.8) 52 (57.2)
**Race *n* (%)** Black Colored Indian White	72 (79.2) 12 (13.2) 1 (1.1) 6 (6.6)
**Indication for transplantation *n* (%)** Biliary atresia Acute liver failure (other causes) Hepatitis A acute liver failure Hepatic sinusoidal obstruction syndrome (HSOS) Wilson's disease Primary hyperoxaluria Hepatoblastoma Adenovirus acute liver failure Alagille syndrome Hepatocellular carcinoma	46 (50.5) 12 (13.2) 12 (13.2) 5 (5.5) 4 (4.4) 4 (4.4) 3 (3.3) 1 (1.1) 1 (1.1) 1 (1.1)
Combined liver/renal transplant *n* (%)	1 (1.1)
**Type of liver transplant *n* (%)** Related living Donors Split Whole	49 (53.8) 29 (31.9) 13 (14.3)

*Note:* Split and whole grafts were from deceased donors.

Abbreviations: IQR, interquartile range; *N*, number of patients.

### 3.2 Incidence and Risk Factors for Acute Cellular Rejection and Plasma Cell‐Rich Rejection

Following LT, ACR occurred in 45 of the 91 patients (49.5%), and 14 of the 45 recipients with ACR (31.1%) subsequently developed PCRR (Table 2). The PCRR cohort comprised 14 PLT recipients, 11 females and 3 males. Most of the children who developed PCRR were Black (Table 3). Age at transplantation ranged from 9.2 months to 139.8 months, with a median age of 32.2 months (Table 3). Although higher MLVI scores were observed among recipients with ACR overall, MLVI did not differ significantly between PCRR and non‐PCRR groups. Suboptimal adherence to immunosuppression, as evidenced by high MLVI scores, was associated with ACR.

**TABLE 2:** Clinical characteristics of study patients.

Characteristic	Overall (*n* = 91) *N* (%)	PCRR (*n* = 14) *N* (%)	No PCRR (*n* = 77) *N* (%)
Acute Cellular Rejection	45 (49.5)	14 (100)	31 (40.3)
Late ACR (> 6 months)	19 (20.9)	—	—
Time to LAR (months), median (IQR)	20.3 (12.6–22.4)	—	—
Plasma Cell‐Rich Rejection	14 (15.3)	14 (100)	—
Time to PCRR (months), median (IQR)	—	20.6 (7.5–28)	—
Chronic Rejection	10 (11.0)	5 (35.7)	5 (6.5)
Time to CR (months), mean ± SD	43.2 ± 40.5	66.3 ± 38.9	53.4 ± 28.3
Alive at last follow‐up	65 (71.4)	11 (78.6)	54 (70.1)
Mortality	28 (30.8)	3 (21.4)	25 (32.5)
C4d positive	4 (4.4)	3 (21.4)	1 (3.2)
ANA positive	5 (5.5)	5 (35.7)	0
Central perivenulitis (CP)	13 (14.3)	12 (85.7)	1 (3.2)
**Rejection episodes**			
One episode	14 (15.4)	1 (7.1)	12 (15.6)
Two episodes	11 (12.1)	5 (35.7)	6 (7.8)
Three episodes	13 (14.3)	8 (57.1)	5 (6.5)
**Banff (RAI) prior to PCRR**			
Mild	5 (5.5)	1 (7.1)	5 (6.5)
Moderate	11 (12.1)	4 (28.6)	7 (9.1)
Severe	13 (14.3)	9 (64.3)	14 (18.2)

*Note:* Values are presented as numbers (%) or mean ± SD or median IQR unless indicated otherwise.

Abbreviations: ACR, acute cellular rejection; CR, chronic rejection; IQR, interquartile range; LAR, late acute rejection; PCRR, plasma cell‐rich rejection; RAI, rejection activity index; SD, standard deviation.

**TABLE 3:** Clinical and histopathological characteristics of pediatric liver transplant recipients with plasma cell‐rich rejection.

Pt	Sex	Race	Age (months)	Dx	LT type	CMV	ANA	C4d	CP	CR	Histopathology/complications	MLVI	Outcome
1	F	W	24.3	BA	Split	LDL	Positive (1:80)	Neg	No	No	Bridging fibrosis; plasma cell infiltrate; Banff RAI score 6; Biliary stricture	1.0	Alive
2	F	B	51.5	HSOS	Split	LDL	Positive (1:2560)	Pos	Yes	No	Endothelialitis; plasma cell infiltrate; Banff RAI score 5	1.1	Alive
3	F	B	54.9	HSOS	Split	VL: 98 510	Positive (1:160)	Pos	Yes	No	Plasma cell infiltrate; portal fibrosis; Biliary stricture	2.7	Alive
4	F	B	30.3	BA	RLD	LDL	Negative	Neg	Yes	No	Plasma cell infiltrate; Biliary stricture	4.0	Alive
5	F	B	139.8	BA	Split	LDL	Positive (1:80)	Neg	Yes	No	Plasma cell infiltrate 45%	3.5	Dead
6	F	B	27.6	BA	RLD	VL:15985	Negative	Neg	Yes	Yes	Plasma cell infiltrate; Biliary stricture PV thrombosis	0.8	Alive
7	F	C	16.3	BA	RLD	VL: 960	Negative	Neg	No	No	Plasma cell infiltrate with clustering	0.7	Dead
8	M	C	34.1	ALF‐HAV	Split	VL: 2252	Negative	Neg	Yes	Yes	Plasma cell infiltrate, Banff RAI score 6; Biliary stricture	1.6	Alive
9	F	W	9.2	ALF	RLD	LDL	Negative	Neg	Yes	No	Portal infiltration of plasma cells	1.2	Alive
10	F	B	98	ALF	Reduced	LDL	Negative	Neg	Yes	No	Plasma cell infiltrate, Endothelialitis, Banff RAI score 7	2.7	Alive
11	M	B	15.2	BA	RLD	LDL	Negative	Neg	Yes	No	Plasma cell infiltrate	1.5	Alive
12	F	B	27.4	BA	Split	LDL	Positive (1:80)	Neg	Yes	Yes	Plasma cell infiltrate	3.3	Alive
13	M	B	107	ALF‐HAV	RLD	LDL	Negative	Neg	Yes	Yes	Isolated CP, plasma cell infiltrate	2.2	Alive
14	F	B	58.6	ALF‐HAV	Split	LDL	Negative	Pos	Yes	Yes	Portal infiltrate of plasma cells, interface activity, Banff RAI score 7	1.4	Dead

Abbreviations: ALF, acute liver failure; ANA, antinuclear antibody; B, black; BA, biliary atresia; CP, central perivenulitis; CR, chronic rejection; HAV, hepatitis A virus; HSOS, hepatic sinusoidal obstruction syndrome; LDL, lower than detectable; MLVI, medication level variability index; PV, portal vein; RLD, related living donor; VL, viral load; W, white.

PCRR had a median time to onset of 20.6 months (IQR 7.5–28) and was significantly associated with more than three prior episodes of ACR (*p* < 0.001) and autoimmune seropositivity (*p* = 0.008). Patients with PCRR were also more likely to develop severe ACR episodes (Banff Rejection Activity Index score ≥ 7) compared to those without PCRR (*p* < 0.001, Table 4).

**TABLE 4:** Comparison of clinical and histopathological features among plasma cell‐rich rejection, acute cellular rejection only, and no rejection groups.

Characteristic	PCRR (*n* = 14, %)	ACR only (*n* = 31, %)	No rejection (*n* = 46, %)	*p*
ANA positive, *n* (%)	5 (35.7)	0	NT	0.008
C4d positive, *n* (%)	3 (21.4)	1 (3.2)	0	0.429
≥ 3 Rejection Episodes, *n* (%)	8 (57.1)	5 (16.1)	—	< 0.001
Central Perivenulitis, *n* (%)	12 (85.7)	1 (3.2)	—	< 0.001
Chronic Rejection, *n* (%)	5 (35.7)	2 (6.5)	3 (6.5)	0.006
Banff RAI Score 7–9, *n* (%)	9 (64.3)	7 (22.6)	—	< 0.001
MLVI, Median IQR	2.05 (1.2–2.8)	2.05 (1.1–3.3)	1.46 (0.8–2.7)	0.310
CMV disease, *n* (%)	3 (21.4)	6 (19.4)	5 (10.9)	0.910

Abbreviations: ACR, acute cellular rejection; ANA, antinuclear antibody; C4d, complement component 4d; CMV, cytomegalovirus; CR, chronic rejection; MLVI, medication level variability index; NT, ANA not tested in the entire cohort; PCRR, plasma cell‐rich rejection; RAI, rejection activity index.

### 3.3 Histopathological Findings

Histopathologically, PCRR was characterized by prominent plasma cell infiltrates in the portal and perivenular regions in all biopsy specimens, exceeding 30% of the inflammatory infiltrate. Central perivenulitis was present in 85.7% (12/14) of patients with PCRR; CR developed in 35.7% of patients. Endothelialitis was observed in 14.3% of cases, while interface activity, bridging fibrosis, and portal fibrosis were each noted in 7.1% of patients (Table 3). C4d staining was performed in all PCRR cases, of which three (21.4%) were positive. The prevalence of CMV disease was higher in the PCRR group (21.4%) compared to the non‐PCRR group (14.3%), although the difference was not statistically significant (*p* = 0.910, Table 4).

### 3.4 Impact of Plasma Cell‐Rich Rejection on Survival

#### 3.4.1 Chronic Rejection

In adjusted analysis, recipients experiencing more than three rejection episodes had significantly increased odds of developing PCRR (OR 22.04, 95% CI 5.08–95.52; *p* < 0.001, Table A1). MLVI of greater than 2.5 and CMV disease were not independently associated with PCRR, suggesting that recurrent rejection burden may be the dominant driver of the plasma cell‐rich phenotype.

**FIGURE 2:**
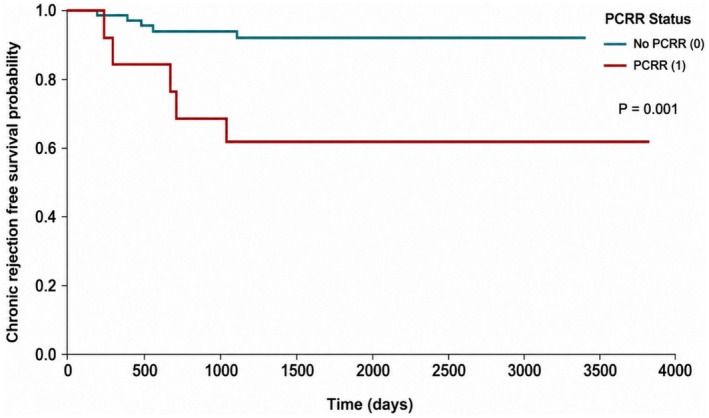
Kaplan–Meier chronic rejection‐free survival according to plasma cell‐rich rejection status.

Chronic rejection developed in 5 of 14 recipients with PCRR (35.7%) compared with 5 of 77 recipients without PCRR (*p* = 0.006). Plasma cell‐rich rejection was independently associated with the development of chronic rejection in a logistic regression analysis (OR 8.00, 95% CI 1.93–33.10; *p* = 0.004; Table A2). The logistic regression model was statistically significant (*p* = 0.005), and Kaplan–Meier analysis demonstrated a propensity to CR in recipients with PCRR compared with those without PCRR (log‐rank *p* = 0.006, Figure 2).

#### 3.4.2 Mortality in Patients With PCRR

**FIGURE 3:**
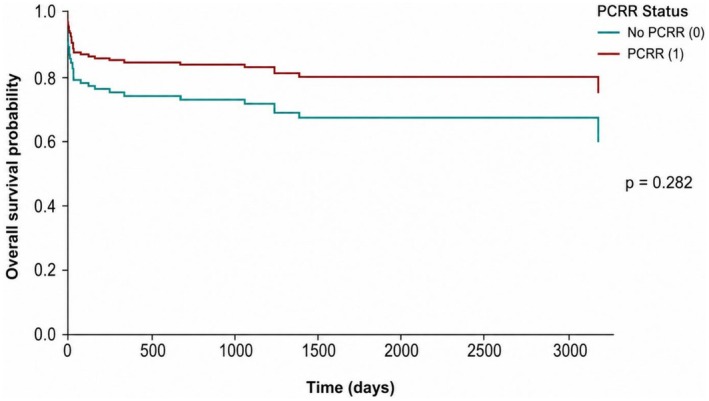
Kaplan–Meier overall survival according to plasma cell‐rich rejection status.

Over the follow‐up period, mortality was observed in 3 of 14 recipients with PCRR (21.4%) and in 25 of 77 recipients without PCRR (32.5%). The Cox proportional hazards model showed that plasma cell‐rich rejection was not associated with mortality (HR 0.51, 95% CI 0.15–1.73; *p* = 0.282, Figure 3).

Severe acute cellular rejection (Banff RAI score 7–9) was associated with increased mortality in the Cox proportional hazards analysis (HR, 1.42; 95% CI, 1.00–1.99; *p* = 0.049). MLVI was independently associated with increased mortality in Cox proportional hazards analysis (HR 1.06, 95% CI 1.03–1.09; *p* < 0.001). CMV disease was independently associated with mortality in Cox proportional hazards analysis (HR 3.71, 95% CI 1.67–8.25; *p* = 0.001, Table A3).

## 4 Discussion

### 4.1 Prevalence and Timing of PCRR in This Cohort

The Banff 2016 update (Demetris et al.) estimates the PCRR to be 3%–5% in PLT recipients. In this retrospective study, PCRR occurred in 15.3% of PLT recipients, a prevalence substantially higher than the Banff estimates [5, 6, 7]. This discrepancy may reflect our cohort's unique characteristics, including a higher prevalence of autoimmune seropositivity, elevated MLVI scores, and longer post‐LT follow‐up, which enabled detection of PCRR at a median of 20.6 months post‐LT [6, 14]. Although PCRR was associated with the development of CR, and the mortality rate in the PCRR group was 21.4% vs. 32.5% in the non‐PCRR group, we did not demonstrate a statistically significant association between PCRR and mortality.

### 4.2 Relationship Between ACR and PCRR

Previous work supports the notion that recurrent ACR increases the severity of the alloimmune response, leading to plasma cell infiltration and thus predisposing to PCRR, with inadequate immunosuppression and poor adherence recognized as drivers of ACR and LAR [6, 8, 11]. In our cohort, multiple ACR episodes were associated with the development of PCRR; all patients who developed PCRR had experienced at least one prior ACR episode. Inadequate immunosuppression and poor adherence further contributed to the development of ACR, as evidenced by elevated MLVI scores [6, 9].

Ozturk et al. reported that chronic viral infections, CMV in particular, may stimulate plasma cell proliferation, contributing to the development of PCRR [7]. PCRR is a multifactorial process involving alloimmune injury, inadequate immunosuppression, and infections [6, 7, 9].

### 4.3 Histopathological Features of PCRR

Histopathologically, central perivenulitis is considered a hallmark of PCRR, reflecting immune‐mediated damage and an alloimmune response to the donor liver [15, 16]. This reaction is often a result of inadequate immunosuppression or ACR [15]. Central perivenulitis is typically associated with ductopenia, zone 3 fibrosis, and plasma cell‐rich infiltrates, and when left untreated, may result in hepatocyte dropout and progression to CR [15, 16]. Treatment of PCRR typically involves intensification of immunosuppressive therapy [7, 16, 17]. In our cohort, central perivenulitis was a dominant histological feature of PCRR [15, 16].

### 4.4 C4d Staining Patterns in PCRR

AMR is associated with strong C4d positivity due to classical complement activation by DSAs [4, 5, 17, 18]. However, C4d interpretation can be technically challenging, with the potential for false‐positive and false‐negative results due to variability in fixation, staining techniques, and sampling errors. Consequently, a negative C4d result should be interpreted cautiously and within the full clinical and histopathological context. In this cohort, C4d staining was positive in 21% of patients who underwent C4d testing, indicating that the majority were C4d‐negative. These histological findings highlight the immune complex‐ and possibly autoimmune‐driven nature of PCRR, with overlap among ACR, AMR, and chronic graft injury [14]. The predominance of C4d‐negative PCRR in our cohort suggests that non‐complement‐activating antibodies or T‐cell‐ and plasma‐cell‐mediated mechanisms may be central to its pathophysiology [7, 16, 17]. This supports the view that C4d‐negative PCRR may represent a distinct rejection phenotype, complicating the distinction between AMR and ACR [4, 5, 7]. Despite diagnostic uncertainty, C4d‐negative PCRR appears to be clinically significant in our cohort [7].

### 4.5 Autoantibodies and Autoimmune Overlap

Previous studies have shown that PCRR is often associated with positive autoantibodies [14]. Although the presence of ANA is not required for the diagnosis of PCRR, persistent ANA positivity has been associated with CR [7, 14]. The exact mechanism remains unclear but may relate to post‐transplantation immune stress, drug exposure, viral infections, or unmasking of latent autoimmune conditions [19]. In this cohort, PCRR was frequently associated with positive autoantibodies. This pattern suggests that, in our population, PCRR may involve not only alloimmune injury but also an autoimmune component or overlap with AIH‐like processes [14, 19].

### 4.6 Adherence and MLVI

Non‐adherence to immunosuppressive therapy is associated with LAR, CR, and increased mortality [10, 20]. The MLVI is a valuable surrogate marker for assessing adherence to immunosuppressive therapy; however, its accuracy is dependent on the consistency of medication administration and the timing of blood sampling. While non‐adherence remains a principal driver of elevated MLVI, tacrolimus metabolism, drug side effects, and drug interactions must also be considered and require further elucidation [21]. Tacrolimus metabolism varies between patients, contributing to variability in tacrolimus levels. Genetic polymorphisms, particularly CYP3A5 expression, are prevalent in individuals of African descent and are associated with increased tacrolimus clearance, resulting in lower trough concentrations and increased drug variability [22].

Shemesh et al. highlighted a high incidence of LAR (53%) in patients with an MLVI > 2.5, which is predictive of poor graft survival [10, 20, 21, 23].

In our cohort, the ACR rate was higher than that reported in other centers and was associated with elevated MLVI scores. Although a direct relationship between MLVI and PCRR was not established in our study, higher MLVI scores were observed in patients with ACR, suggesting that fluctuating immunosuppressive levels may have contributed to the occurrence and severity of ACR episodes and increased the likelihood of PCRR development [10, 16, 17]. Poor adherence to immunosuppression among our PLT recipients was associated with ACR, LAR, and CR. However, the specific factors contributing to medication non‐adherence were not assessed and warrant further study. Recurrent or severe episodes may predispose patients to the development of PCRR and CR, highlighting the importance of close monitoring and optimization of immunosuppressive therapy [7].

### 4.7 Outcomes: PCRR, CR, and Survival

PCRR was not significantly associated with reduced post‐transplant survival. However, it was significantly associated with the development of chronic rejection, with higher CR rates observed in the PCRR group compared to the non‐PCRR group. These findings align with previous reports suggesting that PCRR, particularly when it progresses to CR, represents a severe form of alloimmune injury [7].

Multivariate analysis identified severe rejection episodes (Banff Rejection Activity Index score 7–9), elevated MLVI scores, and CMV disease as factors associated with mortality in our study. Patients followed a uniform CMV prevention and monitoring protocol. Individuals classified as high‐risk received valganciclovir prophylaxis for three months, followed by regular CMV viral load testing, while those in intermediate or low‐risk categories were managed with acyclovir prophylaxis and clinical observation. Despite these measures, CMV disease remained a notable contributor to mortality in this study [24].

Improving adherence, enhancing early detection of rejection, and individualizing immunosuppression are crucial steps in preventing PCRR and improving outcomes.

## 5 Conclusions

We found that PCRR in PLT recipients was more prevalent than in earlier studies and was associated with recurrent and severe acute cellular rejection episodes, autoimmune seropositivity, and central perivenulitis on histopathology in this cohort. These findings suggest that PCRR may represent a spectrum of immune‐related injury ranging from ACR to autoimmunity. PCRR was independently associated with the development of chronic rejection but not with mortality. Poor adherence, severe rejection episodes, and CMV disease contributed to mortality in this cohort. These findings emphasize the importance of early recognition and management of ACR, optimization of immunosuppression, and early identification of PCRR to prevent progression to CR.

## 6 Limitations and Future Directions

This study's retrospective design and small sample size limit generalizability and statistical power, particularly for detecting associations between PCRR and mortality. The absence of routine C4d staining and incomplete DSA testing may have led to underdiagnosis of antibody‐mediated rejection, limiting comprehensive immunological characterization of rejection phenotypes. MLVI was used as a surrogate marker for adherence, but direct measures of psychosocial and socioeconomic determinants were not evaluated and may have contributed to immunosuppressive variability. Future prospective multicenter studies incorporating standardized immunological testing, protocol biopsies, and validated adherence measures are needed to clarify the pathogenesis of PCRR, improve diagnostic accuracy, and develop targeted interventions to prevent progression to CR and graft failure.

## Funding

The authors have nothing to report.

## Disclosure

The authors have nothing to report.

## Ethics Statement

The study was reviewed and approved by the University of Witwatersrand Human Research Ethics Committee (HREC number: M2211108).

## Conflicts of Interest

The authors declare no conflicts of interest.

## Supporting information


**Table S1:** List of definitions used in the study.
**Table S2:** List of abbreviations.

## Data Availability

The data that support the findings of this study are available from the corresponding author upon reasonable request.

## Appendix A

**TABLE A1:** Key predictors for PCRR in the multivariate logistic regression.

Variable	OR	95% CI	*p*
Rejection episodes > 3	22.04	5.08–95.52	< 0.001
MLVI > 2.5	0.54	0.12–2.35	0.412
Banff Rejection Activity Index (RAI) score 7–9	3.60	1.66–7.78	0.001

Abbreviations: Banff RAI score 7–9, severe acute cellular rejection (Rejection Activity Index score 7–9); CI, confidence interval; MLVI, medication level variability index; OR, odds ratio; PCRR, plasma cell‐rich rejection.

**TABLE A2:** Key predictors of chronic rejection in the multivariate logistic regression.

Variable	OR	95% CI	*p*
PCRR	8.00	1.93–33.10	0.004

Abbreviations: CI, confidence interval; CR, chronic rejection; OR, odds ratio; PCRR, plasma cell‐rich rejection.

**TABLE A3:** Cox proportional hazards analysis of predictors of mortality.

Variable	HR	95% CI	*p*
Plasma cell‐rich rejection	0.51	0.15–1.73	0.282
MLVI	1.06	1.03–1.09	< 0.001
CMV disease	3.71	1.67–8.25	0.001
Banff RAI score 7–9	1.42	1.00–1.99	0.049

Abbreviations: Banff RAI score 7–9, severe acute cellular rejection (Rejection Activity Index score 7–9); CI, confidence interval; CMV, cytomegalovirus; HR, hazard ratio; MLVI, medication level variability index.
