# Supplementary material for: Clinicopathological Associations of Plasma Cell‐Rich Rejection in Pediatric Liver Transplant Recipients
Source: Pediatr Transplant. 2026 Jul 14;30(7):e70403. doi: 10.1111/petr.70403 (PMC13366277; doi:10.1111/petr.70403)
Supplement: Supplementary file 1 — Table S1: List of definitions used in the study. Table S2: List of abbreviations. [file PETR-30-e70403-s001.docx]

Supplementary Material:

Supplementary Table S1: List of definitions used in the study

| **Term** | **Definition** |
| --- | --- |
| **Acute cellular rejection** | A T cell-mediated immune response targeting the liver allograft, assessed using the Rejection Activity Index (RAI), which evaluates portal inflammation, bile duct injury, and venous endothelial inflammation. ¹ |
| **Antibody-mediated rejection** | A form of graft injury associated with donor-specific antibodies (DSAs), characterised histologically by endothelial injury, portal capillaritis, and C4d deposition. ¹ |
| **Antinuclear antibody positivity** | Defined as an antinuclear antibody titre of ≥1:160 by indirect immunofluorescence. |
| **C4d staining** | An immunohistochemical marker of classical complement activation used to support the diagnosis of antibody-mediated rejection (AMR) and mixed rejection phenotypes such as plasma cell-rich rejection (PCRR). C4d immunohistochemistry was performed on formalin-fixed, paraffin-embedded liver biopsy tissue using standard automated techniques with diaminobenzidine (DAB) as the chromogen. Staining was assessed semi-quantitatively according to the proportion of portal microvasculature demonstrating linear endothelial staining (0 = none; 1+ = <10%; 2+ = 10–50%; 3+ = >50%). A score of ≥2+ was considered positive.¹˒² |
| **CMV infection** | Presence of detectable cytomegalovirus (CMV) DNAemia with a viral load >1000 IU/mL in the absence of clinical symptoms or histological evidence of tissue-invasive disease. Histological evidence was defined as viral cytopathic inclusions on routine haematoxylin and eosin staining and/or positive CMV immunohistochemistry.³ |
| **CMV disease** | CMV infection accompanied by clinical features consistent with end-organ involvement.³ |
| **Central perivenulitis** | A histopathological feature characterised by inflammation and hepatocyte dropout surrounding central veins, often accompanied by red blood cell extravasation and varying degrees of hepatocellular injury.⁴ |
| **Chronic rejection** | Defined according to the Banff 2016 criteria as bile duct loss affecting ≥50% of portal tracts and/or obliterative arteriopathy, with associated portal or bridging fibrosis. Diagnosis was based primarily on histopathological findings, supported by biochemical evidence of graft dysfunction where available.¹ |
| **Donor-specific antibodies** | Circulating antibodies directed against donor human leukocyte antigen (HLA), implicated in the development of antibody-mediated rejection and associated with graft dysfunction and reduced graft survival.² |
| **Late acute rejection** | Acute rejection occurring more than six months after transplantation, sharing histological features with early acute cellular rejection but often presenting with more advanced clinical and pathological features.⁵ |
| **Medication level variability index** | A quantitative measure of fluctuations in trough immunosuppressant drug concentrations over time. An MLVI >2.5 is considered indicative of poor medication adherence and is associated with an increased risk of rejection.⁶ |
| **Plasma cell-rich rejection** | Defined by plasma cells comprising >30% of the inflammatory infiltrate within portal or perivenular regions, frequently accompanied by interface hepatitis and/or central necro-inflammatory activity involving the majority of portal tracts or central veins.¹ |

Supplementary Table S2. List of abbreviations

| **Abbreviation** | **Definition** |
| --- | --- |
| ACR | Acute cellular rejection |
| AIH | Autoimmune hepatitis |
| AMR | Antibody-mediated rejection |
| CP | Central perivenulitis |
| CMJAH | Charlotte Maxeke Johannesburg Academic Hospital |
| CMV | Cytomegalovirus |
| CNIs | Calcineurin inhibitors |
| DSA | Donor-specific antibodies |
| EBV | Epstein–Barr virus |
| HREC | Human Research Ethics Committee |
| LAR | Late acute rejection* |
| LT | Liver transplantation |
| MLVI | Medication level variability index |
| PCRR | Plasma cell-rich rejection |
| PLT | Paediatric liver transplantation** |
| RAI | Rejection activity index |
| RLD | Related living donor |
| SA | South Africa |
| TCMR | T cell-mediated rejection |
| WDGMC | Wits Donald Gordon Medical Centre |

References:

1. Demetris AJ, Bellamy C, Hübscher SG, et al. 2016 comprehensive update of the Banff working group on liver allograft pathology: Introduction of antibody-mediated rejection. *Am J Transplant*. 2016;16(10):2816-2835. doi:10.1111/ajt.13909

2. Ozturk NB, Schiano TD, Fiel MI. Histologic and Clinical Outcomes of Patients Developing Post-Liver Transplant Plasma Cell-Rich Rejection. *Am J Clin Pathol*. 2023;160(1):49-57. doi:10.1093/ajcp/aqad004

3. Walabh P, Moore DP, Paget G, et al. Healthcare disparity and its associations with cytomegalovirus disease in pediatric liver transplant recipients in South Africa. *Transpl Infect Dis*. 2022;24(6):1-11. doi:10.1111/tid.13917

4. Wu TT, Abraham SC, Freese DK, Ishitani MB, Krasinskas AM. Significance of central perivenulitis in pediatric liver transplantation. *Am J Surg Pathol*. 2008;32(10):1479-1488. doi:10.1097/PAS.0b013e31817a8e96

5. Bateman J, Anugwom C, Zhou Y, Lim N, Adeyi O. Late-Onset Rejection in Liver Allograft Biopsies: An Analysis of Process, Pattern, and Clinical Implications. *Am J Clin Pathol*. 2023;159(3):283-292. doi:10.1093/ajcp/aqac162

6. Shemesh E, Bucuvalas JC, Anand R, et al. The Medication Level Variability Index (MLVI) Predicts Poor Liver Transplant Outcomes: A Prospective Multi-Site Study. *Am J Transplant*. 2017;17(10):2668-2678. doi:10.1111/ajt.14276
